# Long-Term Hydromethylthionine Treatment Is Associated with Delayed Clinical Onset and Slowing of Cerebral Atrophy in a Pre-Symptomatic P301S *MAPT* Mutation Carrier

**DOI:** 10.3233/JAD-210390

**Published:** 2021-09-28

**Authors:** Peter Bentham, Roger T. Staff, Bjoern O. Schelter, Helen Shiells, Charles R. Harrington, Claude M. Wischik

**Affiliations:** a TauRx Therapeutics Ltd., Aberdeen, UK; b Aberdeen Royal Infirmary, NHS Grampian, Aberdeen, UK; c Institute for Complex Systems and Mathematical Biology, University of Aberdeen, Aberdeen, UK; d School of Medicine, Medical Sciences and Nutrition, University of Aberdeen, Foresterhill, Aberdeen, UK

**Keywords:** Frontotemporal dementia, hydromethylthionine, leucomethylthioninium, microtubule-associated protein tau, P301S

## Abstract

One of the mutations in the microtubule-associated protein tau, P301S, is causative for dominantly inherited frontotemporal dementia characterized by extensive tau pathology for which no licensed treatment is available. Hydromethylthionine is a potent tau aggregation inhibitor. We report treatment of an asymptomatic carrier of the P301S mutation using hydromethylthionine over a 5-year period beginning at the mean age of onset of clinical decline in the family. During the period of treatment, the rates of progression of cerebral atrophy were reduced by 61%–66% in frontal and temporal lobes, and the patient remained clinically asymptomatic.

## INTRODUCTION

One of the mutations in the microtubule-associated protein tau (*MAPT*) gene in frontotemporal dementia (FTD) occurs in exon 10 (g123789 C > T) which leads to a substitution of proline at position 301 by serine (P301S) [1]. A worldwide cohort study of age at symptom onset, disease duration, and death in genetic FTD identified 20 P301S cases from 5 families [2]. The mean ages of clinical onset and death were 34.3 (N = 15) and 40.8 (N = 10) years, respectively. At clinical presentation, structural imaging shows various combinations of frontotemporal and parietal lobe atrophy with ventricular enlargement [1, 3–6]. Perfusion and metabolic deficits are reported in similar areas and also in the thalamus. For cases with *MAPT* mutations, structural imaging changes can be identified 5–10 years before expected symptom onset, occurring first in the temporal and medial temporal lobes (hippocampus and amygdala) [7]. The rate of temporal atrophy increases as symptoms develop, followed by atrophy of the frontal and parietal lobes [8], and progresses more rapidly than in Alzheimer’s disease (AD) [9, 10].

There are currently no licensed treatments for any form of FTD. Hydromethylthionine mesylate (also known as leuco-methylthionine bis(hydromesylate); LMTM) is being developed by TauRx Therapeutics for treatment of AD [11, 12] and behavioral variant FTD (bvFTD) [13]. The reduced LMT form of methylthionine has been shown to be the active species blocking tau aggregation *in vitro* [14], with a site of action within the proteolytically stable core tau unit of the tau aggregates found in both AD and FTD [15–17]. The oxidized MT^+^ species present in methylthioninium chloride (MTC) requires conversion to the leucomethylthioninium (LMT) form to permit cell uptake [18]. LMTM was tested in a Phase III clinical trial in 220 patients with behavioral variant FTD in which 200 mg/day was compared with 8 mg/day intended as an inactive control for urinary discoloration. A *post-hoc* population pharmacokinetic (PK) analysis in 171 of the 220 subjects revealed a steep exposure-response relationship in the group receiving the 8 mg/day dose and no additional benefit at 200 mg/day [13]. The majority (65%) of patients receiving 8 mg/day had steady-state plasma levels above a threshold of 0.346 ng/ml, defined on the basis of the lower calibration limit of the assay. These had significantly less cognitive and functional decline and reduced brain atrophy over 12 months when compared to the 35% of patients with low plasma levels at the same dose [13]. Essentially identical results were found in two similarly designed trials in AD [19]. Therefore, 8 mg/day is the minimum effective dose of LMTM in both AD and bvFTD [12, 20], although the predicted optimum doses differ: 16 mg/day in AD and 20–60 mg/day in bvFTD. By contrast, the minimum effective dose using MTC was found to be 138 mg/day in a Phase II trial in AD [21]. This dose of MTC was used to treat a 46-year-old patient with sporadic bvFTD [22]. The authors reported clinically relevant, sustained improvements in behavior, motor abilities, and function over a 6-month period. The present study reports the successful treatment of an asymptomatic carrier of the P301S mutation using LMTM over a period of 5 years beginning at the mean age of onset of clinical decline in the family.

## CLINICAL AND LMTM TREATMENT DATA

A 32-year-old male was assessed in a UK teaching hospital having tested positive for the *MAPT* mutation c.901C > T (g123789 C > T) in exon 10 (CCG to TCG) (Pro301Ser) (Medical Genetics Service Molecular Genetics Laboratory, Addenbrooke’s Hospital Cambridge). The known family history spans four generations and includes 10 cases. The mean age of clinical onset for this family is 38.4 years (N = 5) and the mean age of death is 40.4 years (N = 8). At initial assessment, he had no cognitive complaints but was depressed about the positive test result and was treated with fluoxetine. The informant history described irritability but no other symptoms suggestive of bvFTD. There were no neurological abnormalities. On the Addenbrooke’s Cognitive Examination-Revised (ACE-R) he scored 89 with poor phonemic fluency. On psychometric assessment WTAR estimated IQ was 76, consistent with his longstanding difficulties with reading ability. The WASI FSIQ was average (107). WMS-III index scores ranged from 89 (immediate memory) to 99 (working memory). On tests more specific for frontal lobe function, performance was either average or above average (Hayling verbal suppression 7; Brixton spatial anticipation 6, FAS 34). His MRI scan at that time showed no significant brain abnormality.

Within 12-months the depressive symptoms resolved, and the subject returned to work as an electrician. Subsequent annual reviews revealed no significant symptoms. Six years later, approaching the age of 38, the subject expressed concern because another family member had developed symptoms at a similar age and declined rapidly. A repeat MRI scan showed no significant abnormality or interval change, whereas the HMPAO-SPECT scan showed significant bilateral fronto-temporal perfusion deficits which were worse on the left.

**Fig. 1:**
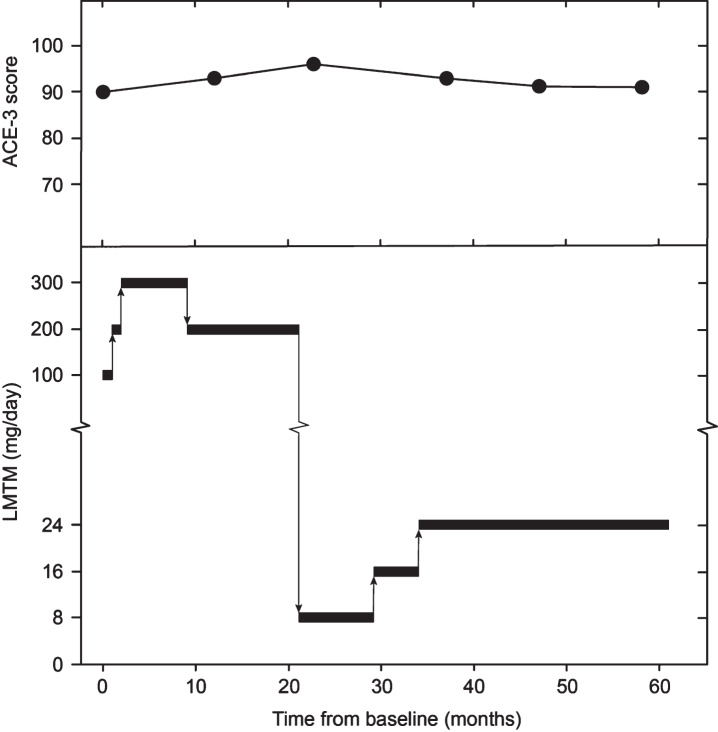
Dosing of LMTM administered to subject and progression of ACE-3 score.

The subject was asymptomatic with no evidence of frontal or temporal atrophy and was therefore ineligible for the ongoing TauRx clinical trial in bvFTD (TRx-237-007; NCT01626378). However, as he had a strong family history, was close to the age of expected symptom onset and had an abnormal HMPAO-SPECT scan, compassionate treatment with LMTM was requested and commenced in January 2016. The subject provided written informed consent for treatment and verbal witnessed consent for anonymized publication of results. Cognition was monitored annually using the ACE-3 [23] and clinical disease severity was assessed using the Frontotemporal Dementia Rating Scale (FRS) [24]. Full blood count including reticulocytes, biochemistry, and vitamins B_12_ and folate were monitored 3-monthly. The doses of LMTM used and the corresponding ACE-3 scores are summarized in Fig. 1. LMTM was well tolerated at all the doses given. Dosing decisions were based on the results emerging from the population PK analyses of data from the bvFTD trial summarized above. Despite commencing treatment at the expected age of clinical onset, there was no decline in the ACE-3 score over the subsequent 61 months of treatment. At month 58, two FRS items were rated as ‘sometimes’ (restlessness and change in food preference), but no behavioral change was evident at interview. At month 61, the informant reported complete resolution. The safety findings were in line with those previously reported in AD and bvFTD studies [11–13], with urinary frequency noted at the 300 mg/day dose, and bilirubin and reticulocyte elevation at both high and low doses.

## NEUROIMAGING DATA

**Fig. 2:**
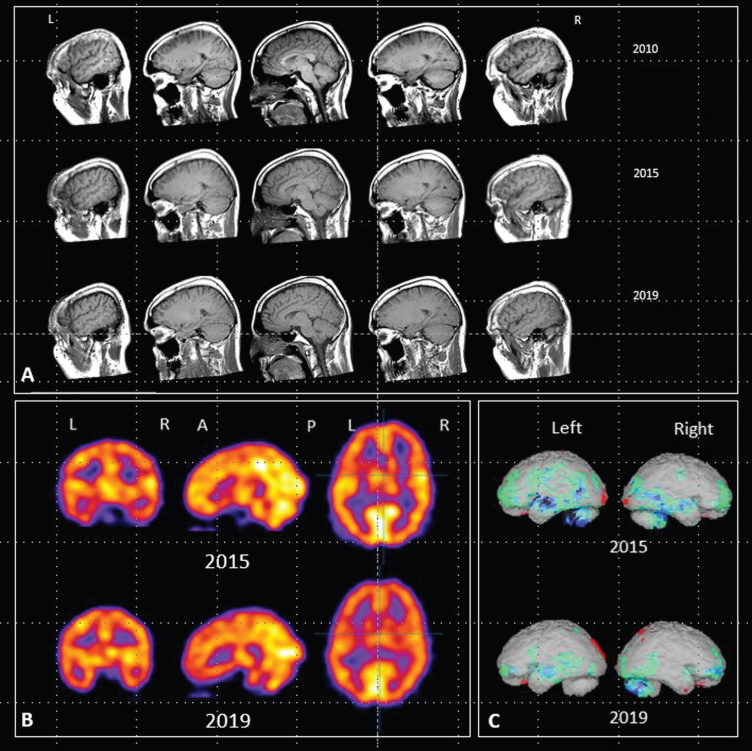
A) Sagittal MRI images acquired in 2010, 2015 and 2019. B) HMPAO-SPECT scan images acquired in 2015 and 2019. C) Rendered images using NeuroGam^TM^ (http://www.segamicorp.com) outputs showing Z scores from the normal range of a control group aged 16 to 45 years with data normalized to cerebellum. Green represents >2 standard deviations below the mean, light blue > 3, dark blue > 4, black > 5. Red represents greater than 2 standard deviations above the mean.

The patient had two MRI scans prior to initiation of LMTM treatment (May 2010 and June 2015) and two further scans in May 2019 and August 2020 while on treatment. HMPAO-SPECT scans were performed in October 2015 and again in July 2019. We used the 2010, 2015, and 2019 MRI images for further analysis because they were of comparable quality and because HMPAO-SPECT scans were also available in 2015 and 2019. There was no change evident between the 2019 and 2020 MRI scans. The three sagittal MRI images are shown in Fig. 2A. These were acquired with three different machines: a Siemens Sonata (2010), a Signa Excite (2015), and a Signa Explorer (2019) all at 1.5 Tesla, using a T1-weighted 2D spin echo sequence with in plane pixel sizes of 0.69, 0.71, and 0.72 mm and slice thicknesses of 6.5, 6.0, and 6.0 mm, and the TR/ET were 449/14, 460/13, and 517/13, respectively. These images were of sufficient quality to permit analysis of atrophy rates in frontal and temporal lobes using an in-house version of the brain boundary shift integral (BBSI) [25] and the ICBM (International Consortium for Brain Mapping) template.

The calculated rates of progression of atrophy per annum are summarized in Table 1 for the 5-year period (2010–2015) when the subject was an asymptomatic carrier and the 4-year period of treatment with LMTM (2015–2019) when he would have been expected to convert to the symptomatic phase of the condition. Compared with the pre-treatment period, treatment with LMTM was associated with a 66% reduction in the frontal lobe atrophy rate (from 0.88%/year to 0.30%/year) and a 61% reduction in the temporal lobe atrophy rate (from –0.73%/year to –0.28%/year). These were compared with atrophy rates expected from non-carriers, and asymptomatic and symptomatic carriers of *MAPT* mutations calculated from the data reported by Chen et al. [8] for subjects of comparable mean age at first scan (36 for non-carriers, 31 for asymptomatic carriers, and 39 for the symptomatic group). The presymptomatic rates are comparable to those reported by Chen et al. Conversion to the symptomatic phase is associated with an increase of +78% (from –0.57%/year to –0.84%/year) in frontal cortex atrophy rate and +72% (from –0.74%/year to –1.42%/year) in temporal cortex atrophy rate (Table 1). By contrast, the rates following treatment with LMTM were similar to those calculated for non-carriers (–0.40%/year in frontal cortex and –0.35%/year in temporal cortex).

**Table 1:** Rates of progression of brain atrophy in frontal and temporal lobes before (2010–2015) and while receiving treatment with LMTM (2015–2019) compared with rates of atrophy calculated from data reported by Chen et al. data [8]

**Subjects**	**Frontal**		**Temporal**	
**Case**	**Lobe (cm^**3**^/y, [rate % /y])**	**Change in rate (%)**	**Lobe (cm^**3**^/year, [rate % /y])**	**Change in rate (%)**
2010–2015	–1.54 [–0.88%]		–0.72 [–0.73%]
2015–2019	–0.52 [–0.30%]	–66.3	–0.28 [–0.28%]	–61.0
**Case**	**Cortex GM (cm^**3**^/y, [rate %/y])**	**Change in rate (%)**	**Cortex GM (cm^**3**^/y, [rate %/y])**	**Change in rate (%)**
2010–2015	–0.85 [–0.86%]		–0.54 [–0.91%]
2015–2019	–0.29 [–0.29%]	–65.9	–0.21 [–0.35%]	–61.1
**Chen et al.**	**Cortex GM (cm^**3**^/y, [rate %/y])**	**Change in rate (%)**	**Cortex GM (cm^**3**^/year, [rate %/y])**	**Change in rate (%)**
Asymptomatic	–0.81 [–0.57%]		–0.94 [–0.74%]
Converter	–1.44 [–0.84%]	+77.8	–1.62 [–1.42%]	+72.3
Non-Carrier	–0.55 [–0.40%]		–0.41 [–0.35%]

Figure 2B also shows the HMPAO-SPECT images from 2015 and 2019. Areas of abnormal perfusion can be seen in frontal and temporal lobes in the 2015 images, particularly on the left. The rendered images (Fig. 2C) show comparisons with the Z scores from the range of a control group aged 16 to 45 years normalized to cerebellum. Both sets of images show that the areas of impaired perfusion seen in 2015 were reduced following treatment with LMTM.

## COMMENTARY

In the context of what is known about this rare form of FTD, it is unexpected that the subject remained in employment, asymptomatic, functionally independent, and cognitively stable into his 44th year. The mean age of symptom onset among carriers in the same family is constant and this association is strongest for *MAPT* mutations [2, 7]. The mean age of symptom onset for the subject’s family is 38.4 years (SD 2.1) and the latest known age of onset with the P301S mutation is 42 years. LMTM treatment was commenced at the age of 38 when conversion to the symptomatic phase would have been expected and when the HMPAO-SPECT scan showed fronto-temporal perfusion deficits.

During the 5-year treatment period with LMTM, cognition measured on the ACE-3 did not decline below the baseline value of 90. Naming, which is known to be affected early in genetic FTD due to *MAPT* mutations [7, 26], remained unimpaired. The FRS items, restlessness & change in food preference, were rated as being sometimes present at one assessment but were not present 3 months later and no behavioral change was evident at the last assessment reported in March 2021.

The rates of atrophy prior to LMTM treatment were similar to those expected from the Chen et al. [8] data for asymptomatic carriers and approximately double the rates observed in non-carriers. Following treatment with LMTM, these were reduced by 61%–66% to rates similar to those seen in non-carriers of comparable age. This contrasts with the increase of 72%–78% that would have been expected after the age of 38 when conversion to the symptomatic phase typically occurs in this family. The reduction in brain atrophy rate was also associated with a reduction in the perfusion deficits that had supported initiation of treatment.

The efficacy of LMTM in this case is supported by reductions in atrophy rates and perfusion deficits and indirect clinical comparisons with this and similar kindreds. Normalization of brain atrophy rates and reduction in progression of functional scan deficits following treatment with LMTM have been reported previously in mild AD [12], and the results are also consistent with those reported recently in a trial in patients with sporadic bvFTD [13]. The present results are obviously limited by the fact that they come from a single case, albeit with consistent clinical and imaging observations over a 10-year period. The findings need to be confirmed in a randomized placebo-controlled trial in larger case series to provide a reliable clinical trial basis for the preventative use of LMTM for carriers of *MAPT* mutations for whom no treatment is currently available.
